# Neural correlates of kinematic features of passive finger movement revealed by univariate and multivariate fMRI analyses

**DOI:** 10.1162/IMAG.a.1083

**Published:** 2026-01-07

**Authors:** Gustavo S.P. Pamplona, James Sulzer, Ewa Beldzik, Olivier Lambercy, Silvio Ionta, Roger Gassert, Jarrod Lewis-Peacock

**Affiliations:** SensoriMotorLab, Jules-Gonin Eye Hospital/Fondation Asile des Aveugles, Department of Ophthalmology/University of Lausanne, Lausanne, Switzerland; Rehabilitation Engineering Laboratory (RELab), Department of Health Sciences and Technology, ETH Zurich, Zurich, Switzerland; Department of Child and Adolescent Psychiatry and Psychotherapy, Psychiatric Hospital, University of Zurich, Zurich, Switzerland; Department of Physical Medicine and Rehabilitation, Case Western Reserve University, Cleveland, OH, United States; Research Laboratory of Electronics, Massachusetts Institute of Technology, Cambridge, MA, United States; Department of Psychology, University of Texas at Austin, Austin, TX, United States

**Keywords:** passive movement, MVPA, univariate analysis, fMRI, finger, kinematic features

## Abstract

Finger movements are associated with a relatively large neural representation. Passive finger movement—externally generated movement without voluntary muscle activity—is a robust approach to investigate the neural representation of kinesthesia and proprioception. While some studies have characterized the neural correlates of passive finger movement, they have relied solely on mass univariate analysis, potentially limiting sensitivity. Additionally, limited consideration has been given to stimulus duration, a factor closely tied to kinematic features such as amplitude and velocity, which more recent modeling approaches account for explicitly. Here, we reanalyzed previously published data using both univariate and multivariate analyses to examine how kinesthesia is represented in the brain in neurotypical subjects across two experiments. Systematic passive stimulation of the fingers was provided using an MR-compatible robot while functional magnetic resonance imaging data were recorded. Our analyses were conducted separately for amplitude, velocity, and direction and adjusted for stimulus duration, thereby controlling for this factor whether or not neural activation scaled with it. We provide a detailed mapping of brain areas related to these kinematic features, including sensorimotor, subcortical, and cerebellar regions. In general, multivariate pattern analysis was more sensitive than the univariate approach in identifying brain regions associated with passive finger movement. Our univariate results showed that activity in sensorimotor and subcortical areas was higher for larger amplitudes and slower velocities, which contrasted with the original study’s findings, likely due to our treatment of stimulus duration as a parametric modulator. A novel result was that sensorimotor areas showed higher activation for extension compared to flexion of passive finger movement. Across kinematic features, a larger neural representation was observed for amplitude and direction than for velocity, suggesting that kinesthesia and proprioception may rely more strongly on displacement than on movement. Whereas univariate analyses are limited in addressing heterogeneity across voxels, our multivariate analyses revealed that a broader set of brain regions carried condition-related information about passive movement, based on distributed voxel activity patterns. Together, these findings extend current knowledge on how the brain represents physical kinematic features of finger movements.

## 1 Introduction

Passive movement entails moving the body part while the individual does not perform active movement. At a neuroscientific level, passive movement of body parts can be employed to study kinesthesia and proprioceptive processes in the brain and to consolidate neuroscientific theories of motor control. The brain correlates of passive movement have been studied extensively using various neuroimaging techniques for several body parts, such as the wrist (Bae et al., 2017), hand (Boscolo Galazzo et al., 2014; Guzzetta et al., 2007; Zhavoronkova et al., 2017), arm (Estévez et al., 2014; Weiller et al., 1996), lower limb (Jaeger et al., 2014), and finger (Dueñas et al., 2018; Lolli et al., 2021; Mima et al., 1999; Nurmi et al., 2018; Sugawara et al., 2016). Hand and fingers have a proportionally large somatotopic representation compared to other body parts (Penfield & Rasmussen, 1950). Passive finger movement commonly elicits responses in the supplementary motor area (SMA), the contralateral postcentral (primary somatosensory cortex – S1) and precentral gyrus (primary motor cortex – M1), and the bilateral secondary somatosensory cortex (S2), thalamus, putamen, and ipsilateral cerebellum (Dueñas et al., 2018; Lolli et al., 2021; Mima et al., 1999; Nurmi et al., 2018; Sugawara et al., 2016). This pattern for passive movement is similar to the one associated with active movement (Boscolo Galazzo et al., 2014; Guzzetta et al., 2007; Weiller et al., 1996), but with a relatively weaker strength of brain activity (Lolli et al., 2019; Mima et al., 1999; Zhavoronkova et al., 2017). Early work on peripheral receptors showed that kinematic features of passive movement are dissociated from each other (McCloskey, 1973; Proske & Gandevia, 2009; Sittig et al., 1985), suggesting that kinematic features (such as amplitude, velocity, and direction) could also be represented differentially in the brain.

Proprioception is especially important for the dexterous movements of the hand and fingers, as it enables precise control and awareness of position without visual guidance. Robotically-driven motion, compared to manual stimulation by an experimenter, offers a systematic way to provide subjects with reproducible and stereotyped passive movement of body parts (Aggogeri et al., 2019). When robotics is used in conjunction with functional magnetic resonance imaging (fMRI) through an adapted and compatible construction (Gassert et al., 2006), one can study the passive finger movement using high-resolution imaging and well-controlled kinesthetic stimulation (Dueñas et al., 2018). However, despite the high spatial resolution of fMRI, the characterization of passive finger movement has so far relied on mass univariate analyses (Boscolo Galazzo et al., 2014; Dueñas et al., 2018; Lolli et al., 2019, 2021; Nurmi et al., 2018), in which voxel-wise activation magnitudes are tested independently across subjects and clusters of reliably active voxels are identified after multiple-comparison correction. Such analyses are informative and reflect mean-level differences in activation but are insensitive to distributed patterns of activity across voxels which can also reflect task-relevant information (Davis et al., 2014). Alternatively, multivariate pattern analysis (MVPA) applied to fMRI offers greater sensitivity for characterizing the neural underpinnings of a given task, stimulation, or behavior (Haxby et al., 2014; Hebart et al., 2015; Kriegeskorte et al., 2006; Norman et al., 2006). MVPA uses machine-learning methods to discriminate between conditions based on patterns carried in distributed voxel activity (Averbeck et al., 2006; Kriegeskorte et al., 2006; Riggall & Postle, 2012). While univariate methods rely on voxelwise correspondence across individuals to identify condition-related signal changes, within-subject MVPA exploits distributed patterns of activity within each individual. Although the analysis is restricted to voxels within a common region of interest, MVPA does not require fine-grained voxelwise alignment across subjects for informative patterns to be detected (Coutanche, 2013; Davis et al., 2014). Consequently, MVPA can reveal subtle condition-related differences that may not be detected by traditional univariate approaches and provides spatially accurate brain maps that extend our understanding of neural functioning across subtle experimental variations.

Employing and comparing univariate and multivariate methods, as well as modeling inherent confounds, may substantially advance our comprehension of brain correlates of passive finger movement. Here, we performed new analysis on a previously recorded dataset (Dueñas et al., 2018). This dataset consisted of high-resolution fMRI data from 41 healthy subjects in two independent experiments while an MRI-compatible robotic system provided finger displacement according to different kinematic features. Using univariate and multivariate techniques, we identified neural responses associated with three different kinematic features (amplitude, velocity, and direction) of passive finger movement. We hypothesized that both univariate and multivariate approaches would exhibit heightened sensitivity to amplitude compared to other kinematic features, reflecting brain responses that rely primarily on the proprioceptive input to interpret passive movement. This hypothesis was motivated by previous findings in active finger movement, where sensorimotor activity (measured with univariate analysis controlling for stimulus duration) was more specific to amplitude than to other kinematic features (Shirinbayan et al., 2019). By providing linear (and not oscillatory) movements experimentally and by using a model that controls for false positive rates regardless of whether brain activation scales with stimulus duration, we provide unique insights on the neural responses to passive finger movement. Together, our complementary univariate results and multivariate results deepen our understanding of the neural correlates of passive finger movement.

## 2 Methods

### 2.1 Participants

We performed new analyses of a previously recorded dataset (Dueñas et al., 2018). The data consisted of anatomical and functional MR images collected at the MRI Center of the Psychiatric Hospital of Zurich, University of Zurich, Switzerland. Data were obtained from 41 right-handed healthy subjects who participated in two experiments, hereafter referred to as Exp 1 and Exp 2 (following the nomenclature of the original publication) (Dueñas et al., 2018). The dataset used for the present study contained only subjects whose head displacement during functional runs was no greater than 3 mm, filtered out in the original study. Nineteen subjects participated in Exp 1 (14 females, 27.8 ± 8.9 years) and 22 subjects (11 females, 25.2 ± 2.9 years) participated in Exp 2. The mean age of the participants was not different between the two experiments (two-sample t-test, p = 0.20). All subjects read and gave written consent for data reuse and received monetary compensation for their participation. The study was approved by the local ethics committee (KEK 2010-0190).

### 2.2 Experimental design

**Fig. 1.**
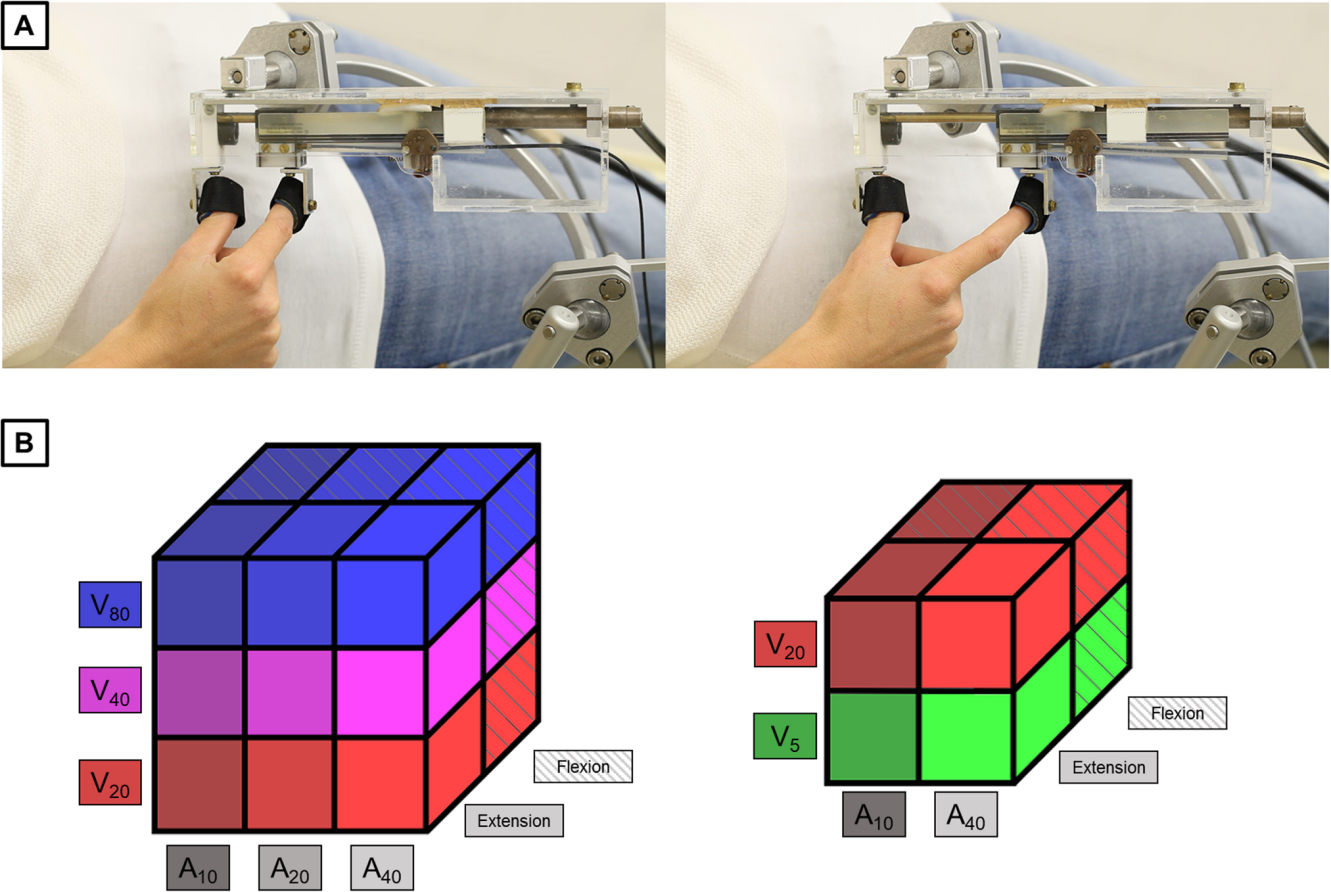
(A) MRI-compatible robot used in Exps 1 and 2. Adduction and abduction of right thumb and index finger relative to each other were provided to the subjects, while they were lying in the MRI scanner, with the wrist positioned in the anatomically neutral position. We show two static positions of the fingers between event-related trials. Relative to a null aperture (Velcro straps touching each other), the left/right image shows an aperture of 20%/80% of the subject’s maximum amplitude, respectively. (B) Factorial designs for Exps 1 and 2, respectively. Each element of the 3-D representation is a possible combination of levels of kinematic features (amplitude, velocity, and direction) in an event-related trial. Exp 1 was designed as a 3 × 3 × 2 factorial design, with three levels of amplitude, three levels of velocity, and two levels of direction. Exp 2 was designed as a 2 × 2 × 2 factorial design, with two levels of amplitude, two levels of velocity, and two levels of direction. Shading represents different levels of amplitude, colors represent different levels of velocity, and textures represent different levels of direction.

We acquired fMRI of the subjects’ brains while they were undergoing robotically-driven passive finger movement, provided through an MRI-compatible robotic system (Fig. 1A). Prior to the MRI recording, we measured the maximum aperture between right thumb and index finger for each subject with a ruler outside the scanner room. Subjects were laid on the scanner table in a supine position with the wrist in the anatomically neutral position. We attached the distal phalanges of the subjects’ right thumb and index finger using Velcro straps to the slave part of the robot (MRI-compatible), previously mounted on the right side of the scanner table. This part of the robot was controlled by the master part (non-MRI-compatible), lying outside the scanner room, and connected to each other through a hydraulic transmission. The robot was designed provide individuals with flexion and extension of the index finger relative to the thumb. The motion of the index finger was controlled during the experiments through an optical encoder. The robotic system has been validated against artifacts for concomitant fMRI recording and poses no safety concerns for participants (Gassert et al., 2006). A dedicated computer with custom code written in LabVIEW (Laboratory Virtual Instrument Engineering Workbench, National Instruments) controlled the robot. Subjects were familiarized with the experimental procedure before being positioned in the MRI scanner. They were instructed to remain relaxed, to not exert active movement in reaction to passive movement, to keep their eyes open, and to not fall asleep during the measurement. Four fMRI runs were acquired for each subject for both Exp 1 and Exp 2.

Each trial (robotically-driven passive movement) consisted of a single displacement of right thumb and index finger relative to each other for a combination of levels of kinematic features—amplitude, velocity, and direction (Fig. 1B). For each trial, we recorded the actual amplitude and duration of the predetermined displacement via sensors, which also allowed us to calculate the trial-specific velocity (amplitude divided by duration). Displacement onsets were also recorded. The order of the trials and the interval between trials were pseudorandomized for each subject prior to scanning. For Exp 1, trials consisted of a 3 × 3 × 2 combination of amplitudes of 10, 20, or 40% of the maximum aperture (A_10_, A_20_, and A_40_), velocities of 20, 40, or 80% of the maximum aperture per second (V_20_, V_40_, and V_80_), and directions of extension or flexion. The interval between trials was 4 ± 2 s. Each unique combination was repeated eight times per run in Exp 1. The run length was 13.5 min for Exp 1. The mean ± standard deviation of the amplitudes in Exp 1 across individuals was 9.6 ± 1.3 mm for A_10_, 18.1 ± 2.6 mm for A_20_, and 37.0 ± 5.3 mm for A_40_. The mean ± standard deviation of the (average) velocities in Exp 1 across individuals was 26.4 ± 3.3 mm/s for V_20_, 56.7 ± 7.1 mm/s for V_40_, and 134.6 ± 17.0 mm/s for V_80_.

For Exp 2, trials consisted of a 2 × 2 × 2 combination of amplitudes of 10 or 40% (A_10_ and A_40_) of the maximum aperture, velocities of 5 or 20% of a maximum aperture per second (V_5_ and V_20_), and directions of extension or flexion. The interval between trials was 12 ± 2 s. Each unique combination was repeated five times per run in Exp 2. The run length was 10.5 min for Exp 2. The mean ± standard deviation of the amplitudes in Exp 2 across individuals was 7.3 ± 1.3 mm for A_10_, and 38.3 ± 4.6 mm for A_40_. The mean ± standard deviation for the (average) velocities in Exp 2 across individuals was 6.8 ± 0.8 mm/s for V_5_, and 30.6 ± 2.6 mm/s for V_20_.

Therefore, Exp 1 utilized a fast event-related design, while Exp 2 employed a slow event-related design. Compared to rapid designs, slow event-related designs allow for better estimation of the hemodynamic response, as there is more time for the blood-oxygenation-level-dependent (BOLD) response to return to the baseline before the onset of the next event. In fast event-related designs, closely spaced trials lead to collinearity, resulting in highly variable estimates. A solution to this issue is to adopt a slow event-related design (De Martino et al., 2008; Mumford et al., 2012), which was the primary reason for conducting Exp 2 in the original study. However, due to the longer intervals between stimuli in slow event-related designs, fewer trials are available compared to rapid designs, making them less efficient for evoking condition-related activity. Consequently, only two levels of amplitudes and velocities were available in Exp 2, whereas three levels were available in Exp 1. Due to these methodological differences, the results from the two experiments are not directly comparable. Nonetheless, we further discuss how differences in outcomes between the experiments may be attributable to these methodological variations.

### 2.3 MRI acquisition

Imaging data were acquired with a Philips Achieva 3 T MR scanner and a 32-channel head coil. For both experiments, we first acquired functional volumes covering the whole cerebrum and the superior part of the cerebellum, obtained with a gradient-echo planar imaging sequence, 35 axial slices, repetition time/echo time (TR/TE) = 2000/35 ms, matrix size = 240 × 240 mm², voxel size = 3 × 3 × 4.07 mm³, 1.1 mm slice gap, and 400 and 306 volumes for Exps 1 and 2, respectively. Next, we acquired high-resolution three-dimensional anatomical images covering the whole brain, obtained with a T1-weighted sequence, 180 sagittal slices, TR/TE = 6.9/3.1 ms, flip angle = 8°, matrix size = 256 × 256 mm², voxel size = 1 mm³). The total duration of a session was about 60 min for each experiment.

### 2.4 Data analysis

#### 2.4.1 MRI preprocessing

All MRI data acquired during passive finger movements were preprocessed prior to statistical mapping analysis, using SPM (Statistical Parametric Mapping, Wellcome Trust Centre for Neuroimaging, www.fil.ion.ucl.ac.uk/spm/) and custom scripts written in MATLAB (Mathworks, Natick, MA). We first slice-time corrected the functional images for each run and subject. We then estimated the six translation and rotation parameters of head motion and used them to spatially realign the functional images to a mean referential image. Next, we coregistered the anatomical image to the mean functional image. Thus, we used the coregistered anatomical image to create a deformation field, which was used to normalize all functional images to the Montreal Neurological Institute (MNI) space. In this process, we resampled the voxel size to 3 mm³. Finally, functional images were spatially smoothed using an isotropic Gaussian kernel of 6 mm³ full-width at half maximum, to be used only for univariate analysis.

#### 2.4.2 Estimation of activation for factorial design

We estimated voxel-wise activation for each unique combination of kinematic feature levels using a general linear model (GLM) for each subject. The estimation process was performed twice: once with unsmoothed functional MR images for MVPA, and once with smoothed images for univariate analysis. For each run, we designed a matrix with 18 regressors (3 amplitude × 3 velocity × 2 direction levels) for Exp 1 and eight regressors (2 amplitude × 2 velocity × 2 direction levels) for Exp 2. Depending on the scientific question, time-on-task effects can act as confounding factors in univariate analysis (Domagalik et al., 2014; Yarkoni et al., 2009). Additionally, stimulus duration can confound MVPA (Todd et al., 2013), potentially causing the classifier to be trained on stimulus duration rather than the condition of interest per se. To account for this confound, we adjusted the activation estimation in run-specific regressors by treating stimulus duration as a covariate of no-interest. Accounting for trial-specific time-on-task differences in the model was first proposed by Grinband et al. (2008), in which the trials were modeled as boxcar functions with varying durations, according to the trial—the so-called variable epoch model. This modeling approach was initially applied in the study by Dueñas et al. (2018), which first described the data used in our present study. However, recent methodological work demonstrates that, while the variable epoch model effectively controls for false positives in cases where neural activity duration scales with time-on-task, it leads to a high rate of false positives when neural activity duration does not scale with time-on task (Mumford et al., 2024). Therefore, a model that minimizes false positives in both scenarios—whether neural activity duration scales or does not scale with time-on-task—is desired. Fulfilling these requirements, a proposed solution is a model uses constant regressors modulated by stimulus duration (Mumford et al., 2024). We adopted this approach to account for stimulus duration to keep the rate of false positives low, regardless of whether activation correlates with stimulus duration. Furthermore, we assumed a full interaction model, in which we accounted for neural activation that could vary with stimulus durations at different levels for each condition (Mumford et al., 2024). Therefore, we modulated each condition-specific constant regressor separately by stimulus duration. These constant regressors were constructed using boxcar functions, based on the onset of each robotic displacement and the mean stimulus duration across all trials within a run for each subject. For each constant regressor, a parametric modulator was added by specifying the mean-centered stimulus duration for each trial within each run. Accordingly, for this model, the regressors of interest and their parametric modulators comprised 36 columns in the design matrix for each run in Exp 1 (3 amplitude levels × 3 velocity levels × 2 directions, plus their modulators) and 16 columns for Exp 2 (2 amplitude levels × 2 velocity levels × 2 directions, plus their modulators). All parametric modulators were modeled as first-order (i.e., linearly associated). Additional regressors representing the covariates of no-interest, namely six head motion parameters and the runs, completed the design matrix. Each regressor was convolved with a canonical hemodynamic response function provided in SPM. We then simultaneously estimated voxel-wise the subject-specific GLM betas for each regressor. For each GLM, simple contrast maps of interest were created using the beta estimates for the constant regressors representing the unique combinations of kinematic feature levels. We used a high-pass filter of 128 s and an auto-regressive model of first degree to remove autocorrelation in the signal. We also applied an individual implicit mask, defining that only voxels with a mean value exceeding 10% of the global signal would be included in the model estimation. This relatively liberal threshold prevents the exclusion of true-positive voxels at the brain’s edges. Any false-positive voxels included by this liberal mask were subsequently excluded through group-level explicit masking (see Section 2.4.3). This analysis underlies the main results presented in Section 3.

As described, we assumed that BOLD signals could potentially relate to stimulus durations at different levels for each condition in our model, which constitutes a full interaction model (Mumford et al., 2024). Therefore, modulated regressors were added to each condition-specific regressor. However, one could also assume that BOLD signals would relate to stimulus durations equally across conditions, without an interaction effect. To explore this possibility further in a complementary analysis, we also designed a model in which a single modulated regressor was added to the design matrix, representing all trials collapsed across conditions. To implement this, we constructed design matrices with condition-specific constant regressors and one condition-unspecific parametric modulator representing all trials within a run. Accordingly, for this non-interaction model, the regressors of interest and the parametric modulator comprised 19 columns in the design matrix for each run in Exp 1 (3 amplitude levels × 3 velocity levels × 2 directions, plus one modulator) and 9 columns for Exp 2 (2 amplitude levels × 2 velocity levels × 2 directions, plus one modulator). All other specification and estimation procedures were identical to those used in the model described above. This estimation was performed for all kinematic features and experiments. This analysis underlies the complementary results presented in Supplementary Figure S7.

#### 2.4.3 Multivariate pattern analysis (MVPA)

We used MVPA to find the brain regions that code kinematic features of passive finger movement, controlled for the influence of the stimulus duration. For the first-level multivariate analysis, we used the activation beta maps for each unique combination of kinematic features within a run obtained for the unsmoothed functional images. This method increases classification performance and power in MVPA and it is a recommended procedure for event-related experiments (Hebart et al., 2015; Mourão-Miranda et al., 2006, 2007; Mumford et al., 2012). Here, we used The Decoding Toolbox (TDT) (Hebart et al., 2015) for MVPA, written for MATLAB and using SPM functions. We used the searchlight approach with an 8-mm-radius sphere, corresponding to 81 voxels. The searchlight approach trains and test a classifier considering all voxels within a roving region-of-interest (ROI) centered on each voxel and assigning the prediction value to that voxel, until all the voxels in the brain are covered. We used a leave-one-run-out cross-validation for the classification analyses, in which we trained a model with three runs (a cross-validation fold) and validated its prediction performance of the condition level in the remaining run. The classification is performed iteratively for all runs and the average performance is assigned to the central voxel of the searchlight ROI, therefore creating a map of cross-validation accuracies based on the local information around each voxel (Kriegeskorte et al., 2006). To avoid overfitting, the classification was performed through a support-vector machine, i.e., by finding an optimal hyperplane constituted by voxels (features) to define the decision boundary between conditions (classes) (Cortes & Vapnik, 1995). The regularization parameter was set to C = 1, a commonly used default, to balance generalization and avoiding overfitting. No scaling method was applied. For each subject, experiment, and kinematic feature, we obtained whole-brain maps of accuracy minus chance (in which the chance was 33.33% (3-class classification) for amplitude and velocity in Exp 1 and 50% (2-class classification) for direction in Exp 1 and for all the conditions in Exp 2). Next, we spatially smoothed the resulting individual maps using a 6-mm isometric Gaussian kernel.

Next, the individual accuracy maps were included in second-level random-effects analyses to compute one-sample t-tests. We thus obtained group-level t-maps in which positive values represent accuracies above chance level for each kinematic feature and experiment. To identify the significant clusters, we used a voxel-wise statistical threshold of p < 0.05, corrected for multiple comparisons using the family-wise error (FWE) method. For Exp 2 and the amplitude condition, we used a voxel-wise statistical threshold of p < 5 × 10^-5^, corrected for multiple comparisons using the FWE method, because the statistical threshold used for other maps resulted in too many significant voxels for this analysis. At the group level, a template-based explicit mask encompassing the entire cerebrum and the superior portion of the cerebellum was applied to eliminate unintended false-positive voxels included by the individually defined implicit masks. Using bspmview (github.com/spunt/bspmview), we generated inflated surface maps and reported peak coordinates (multiple peaks were reported for the same clusters if the separation between them was greater than 20 mm), automatically labeled according to the AAL3 atlas.

#### 2.4.4 Univariate analysis

In parallel, we used univariate analysis to find the brain regions showing differential activity across kinematic features of passive finger movement, controlling for stimulus duration, for both experiments. For this first-level analysis, we estimated the activation beta maps for each unique combination of kinematic features within each run using the smoothed functional images. We then created individual linear combination contrast maps for each subject, replicated and scaled across runs, based on the Kronecker product (using the function ‘spm_make_contrasts’) (Henson, 2015) for a three-way repeated-measures Analysis of Variance (ANOVA).

Next, these individual linear combination contrast maps were included in second-level random-effects analyses to compute group-level maps of the main effects of amplitude, velocity, and direction for both experiments separately. This was done using one-way ANOVAs with unequal variance, constituting an appropriate approach for within-subject designs (Henson, 2015). We obtained group-level F-maps, in which values represent activation estimates greater than the grand mean for each kinematic feature and experiment. To identify significant clusters, we used the same voxel-wise statistical thresholds for each condition and experiment and the same explicit mask as in MVPA (Section 2.4.3). Inflated surface maps, peak coordinates, and region labels were obtained as in the MVPA.

#### 2.4.5 ROI analyses

Results from our whole-brain analyses identified clusters where classification accuracy of activation patterns was significantly above chance for MVPA or where activation differed across conditions for univariate analysis. However, these clusters alone do not allow us to infer condition-specific differences in accuracy or activation estimates within these whole-brain clusters, nor their directionality. Therefore, to better understand condition-specific differences in classification accuracy of activation patterns and activation estimates within these whole-brain clusters, we conducted post-hoc analyses. We centered representative ROIs on the peak coordinates of group accuracy maps for MVPA and group F-value maps for univariate analysis, selecting a maximum of six peaks sorted by the highest accuracy values and labeled according to the Anatomy Toolbox.

In addition, we performed ROI analyses in regions expected to be involved in passive finger movement based on previous literature. These ROIs were predefined and independent of the significance of whole-brain results, serving to test differences in classification accuracy of activation patterns and in mass-univariate activation estimates. Therefore, we defined six ROIs centered on the regions reported by Galazzo and colleagues (Table 2 from Boscolo Galazzo et al. (2014)), that is, the contralateral S1, the SMA, the bilateral S2, the contralateral thalamus, and the ipsilateral cerebellum. More specifically, the ROIs representing the thalamus and the cerebellum are located in the ventral posterolateral nucleus of the thalamus and lobule VI of the cerebellum. The Talairach coordinates provided in the study were converted to MNI space using the function ‘tal2icbm_spm’ (www.brainmap.org/icbm2tal/). The center coordinates are described in Table 1.

**Table 1.** Center coordinates for ROI analyses from regions related to passive finger movement, converted from (Boscolo Galazzo et al., 2014).

	MNI coordinates		
Region label	x	y	z
L S1	-41	-28	61
SMA	-8	-14	62
L S2	-54	-25	20
R S2	54	-27	27
L Thalamus	-17	-20	8
R Cerebellum	25	-49	-26

5-mm-radius spherical ROIs were created with the MarsBaR toolbox (marsbar.sourceforge.net/; (Brett et al., 2002)), corresponding to 19 voxels. For each ROI defined from whole-brain peaks or literature coordinates, we performed MVPA in the voxels within the ROI to determine whether the region could correctly significantly discriminate at least one of the kinematic feature levels. We trained the classifiers with the levels of each kinematic feature of interest. A leave-one-run-out cross-validation was conducted to compute accuracy performance. We generated subject-specific confusion matrices of accuracy performance for each ROI, experiment, and kinematic feature. The accuracy performance for each true/predicted value in the confusion matrix was evaluated for statistical significance using a bootstrapping approach with 1 × 10^5^ resamples (with replacements) of subject-specific accuracy values. We determined whether the performance differed from chance by verifying if the chance level fell outside the confidence interval derived from the bootstrapped distribution. Confusion matrices were presented as transparent if no elements were deemed significant according to this procedure and as non-transparent if at least one element was significant. The confidence interval was calculated based on a significance level of 0.05, adjusted for multiple comparisons dividing it by the number of elements in the confusion matrix (i.e., four for two condition levels and nine for three condition levels). To further evaluate the influence of ROI size and feature scaling on accuracy values, we repeated this analysis using an 8-mm-radius spherical ROI (i.e., 81 voxels) and applied feature scaling across voxels to a range between 0 and 1. The scaling procedure was performed on the training data and subsequently applied to the test data to preserve the independence between training and test sets.

In addition, for each ROI defined from whole-brain peaks or literature coordinates, we performed post-hoc univariate analyses within each ROI to determine whether the region showed significant pairwise differential activation estimates. First, we extracted the average beta of the activation estimates from each unique combination of kinematic features within each ROI. Next, we performed one-way ANOVAs using RStudio (www.rstudio.com/) to compare activation estimates across condition levels for each ROI, experiment, and kinematic feature. For this analysis, we used the function ‘emmeans_test’ (library ‘rstatix’), with a significance level of 0.05 and correction for multiple comparisons according to the Sidak method. Boxplots representing condition-specific distributions were presented as transparent if no pairwise differences were deemed significant according to this procedure and as non-transparent if at least pairwise difference was significant.

The complementary analysis (using a single modulated regressor) was performed only for the univariate ROI analysis, using the ROIs defined from the literature coordinates, because we wanted to determine how the general pattern of results in key regions would be altered by a model controlling for the interaction stimulus duration by condition. This complementary analysis was conducted for all kinematic features and experiments. We extracted the average beta of activation estimates from the conditions of interest (constant regressors) within each ROI for each unique combination of kinematic features. All other procedures for this analysis were identical to the main analysis described above.

#### 2.4.6 Evaluation of MVPA stability across subjects

We additionally analyzed the stability of the MVPA models by classifying state levels across subjects. The stability analysis estimates how generalizable the MVPA results are across subjects. This analysis was done for all ROIs defined from the literature coordinates (section 2.4.5) and for kinematic features and experiments separately. We used the activation estimates for each unique combination of kinematic features determined from the unsmoothed functional images, controlling for the influence of stimulus duration. For each condition level, we trained the model using the condition level estimates for all runs from all subjects minus one and tested it on the remaining subject (leave-one-subject-out cross-validation). No scaling method was applied. We obtained the confusion matrices of the mean accuracies across subjects for each kinematic feature and experiment. Since only one confusion matrix was obtained for the group for this analysis, no statistical testing was applied. The MVPA ROI analysis was considered stable across subjects if the main diagonal was clearly higher than the other values in the resulting confusion matrix.

#### 2.4.7 Comparison of significant voxel counts across conditions for MVPA and univariate analysis

To evaluate how kinematic features differ in terms of discrimination or activation voxel counts, we conducted an additional analysis addressing this question. Specifically, we compared the number of voxels within regions of interest (ROIs) that successfully distinguished between levels of kinematic features (i.e., Amplitude, Velocity, and Direction) across kinematic features, using both MVPA and univariate analyses. For the MVPA, we used the unsmoothed accuracy-minus-chance maps for each participant, obtained as described in Section 2.4.3. Voxels in the subject-specific maps were defined as “significant” when the searchlight analysis centered on that voxel showed an accuracy value at least 10% above chance for each combination of kinematic feature and experiment. This produced thresholded individual MVPA maps indicating the number of searchlights within each ROI that could successfully discriminated across levels of the kinematic features. For the univariate analysis, we used individual smoothed F-maps (for the three-level contrasts) or t-maps (for two-level contrasts), as detailed in Section 2.4.4. Significant voxels were identified using a cluster-forming threshold of uncorrected p = 0.001 and a cluster-level threshold of FDR-corrected p = 0.05. This yielded thresholded individual univariate maps showing the number of voxels exhibiting activation differences across levels of kinematic features. The ROIs for this analysis included those defined in the main ROI analysis (Section 2.4.5)—namely, the left precentral gyrus, left supplementary motor area, right lobule VI of the cerebellum, left ventral posterolateral nucleus of the thalamus, and the left and right secondary somatosensory region—along with the left postcentral gyrus and the left supramarginal gyrus. All ROIs were defined using the AAL3 atlas, with the exception of the secondary somatosensory regions, which are not included in AAL3. These were defined by placing 8-mm-radius spheres centered on the coordinates described in the ROI analysis. For each individual, condition, and experiment, we calculated the number of significant voxels within each ROI for both the MVPA and univariate thresholded maps. Voxel counts across conditions were then compared across kinematic features using generalized linear mixed models for zero-inflated data (using the ‘glmmTMB’ package in RStudio), with separate models run for each ROI and analysis type. A significance level of 0.05 was applied, corrected for multiple comparisons (14 tests: 7 ROIs × 2 analysis types) using the Bonferroni method. Post-hoc pairwise comparisons were performed using estimated marginal means (‘emmeans’ package), with p-values adjusted using the Tukey method.

## 3 Results

### 3.1 Amplitude

#### 3.1.1 Experiment 1

**Fig. 2.**
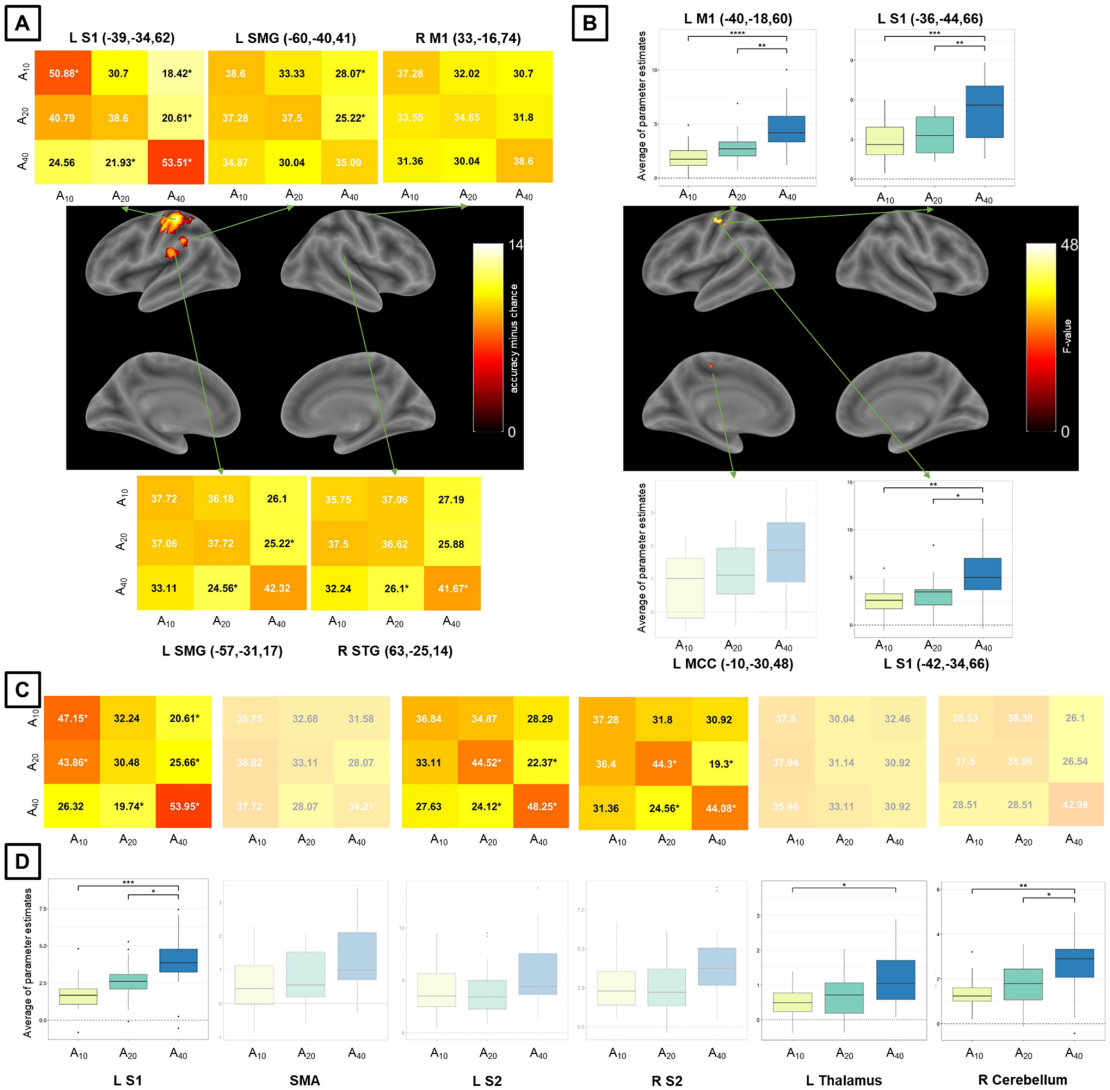
MVPA and univariate analysis results for the amplitude condition and Exp 1. Whole-brain results for (A) MVPA (accuracy significantly above chance) and (B) univariate analysis (significant differential activation), both using a voxel-level inclusion threshold of p < 0.05, FWE (family-wise error)-corrected for multiple comparisons. ROI analysis results (C) for MVPA and (D) univariate analysis. Non-transparent plots in (A) and (C) represent accuracies significantly different from chance. Non-transparent plots in (B) and (D) represent significant main effects of amplitude in activation and post-hoc significant pairwise differences. Asterisks in MVPA results represent accuracies significantly different from chance (33.33%), corrected for multiple comparisons using the Bonferroni method and bootstrapping (10^5^ permutations) across subjects. Asterisks in univariate analysis results represent significant differences across activation estimates, corrected for multiple comparisons (Sidak method – ****p < 0.0001, ***p < 0.001, **p < 0.01, *p < 0.05). For MVPA confusion matrices, numbers in black (white) are higher (lower) than chance. S1/S2 = primary/secondary somatosensory cortex, SMA = supplementary motor area, SMG = supramarginal gyrus, M1 = primary motor cortex, STG = superior temporal gyrus, MCC = midcingulate cortex, L/R = left/right.

For Exp 1 (which included three amplitude levels), whole-brain MVPA revealed a brain network encoding amplitude levels of passive finger movement, which included contralateral S1 and SMG and ipsilateral M1 and STG (Fig. 2A and Table 2). Whole-brain univariate analysis, for the same statistical threshold, showed differential activation across amplitude conditions in the contralateral M1 and S1 and in the MCC (Fig. 2B and Table 2). The activation estimates in these regions increased with amplitude. Overall, whole-brain MVPA was more spatially sensitive than univariate analysis for amplitude of passive movement. MVPA ROI analysis for Exp 1 further showed that, in addition to contralateral S1, the contralateral and ipsilateral S2 also encode amplitude levels (Fig. 2C). For this MVPA ROI analysis in Exp 1, we also report accuracy confusion matrices for amplitude using a larger ROI (Supplementary Fig. S8A) and with scaling (Supplementary Fig. S10A), alongside the original values. Univariate ROI analysis showed that, in addition to contralateral S1, the thalamus and cerebellum showed differential activation across amplitude levels (Fig. 2D), but not bilateral S2. There was no acceptable stability for amplitude in Exp 1 (Supplementary Fig. S1A, top row). Axial slices for whole-brain maps for amplitude in Exp 1 obtained with MVPA and univariate analysis are shown in Supplementary Figure S2.

**Table 2.** Whole-brain MVPA and univariate results for the amplitude condition and Exp 1.

			MNI coordinates		
Region label	Extent (voxels)	Statistics	x	y	z
*MVPA results*					
L postcentral gyrus (L S1)	777	14.47	-39	-34	62
L supramarginal gyrus		10.04	-60	-40	41
L superior temporal gyrus	112	9.54	-57	-31	17
R superior frontal gyrus (R M1)	22	7.68	33	-16	74
R superior temporal gyrus	11	7.47	63	-25	14
*Univariate analysis results*					
Left precentral gyrus (L M1)	165	48.31	-40	-18	60
Left midcingulate cortex (L MCC)	17	44.31	-10	-30	48
Left postcentral gyrus (L S1)	27	42.42	-36	-44	66
Left postcentral gyrus (L S1)	20	38.17	-42	-34	66

Only clusters containing 10 or more voxels are reported. The statistics column corresponds to accuracy minus chance for MVPA results and to F-value to univariate analysis results. Brain regions were labeled through the Anatomy Toolbox and after visual inspection (between parentheses).

#### 3.1.2 Experiment 2

**Fig. 3.**
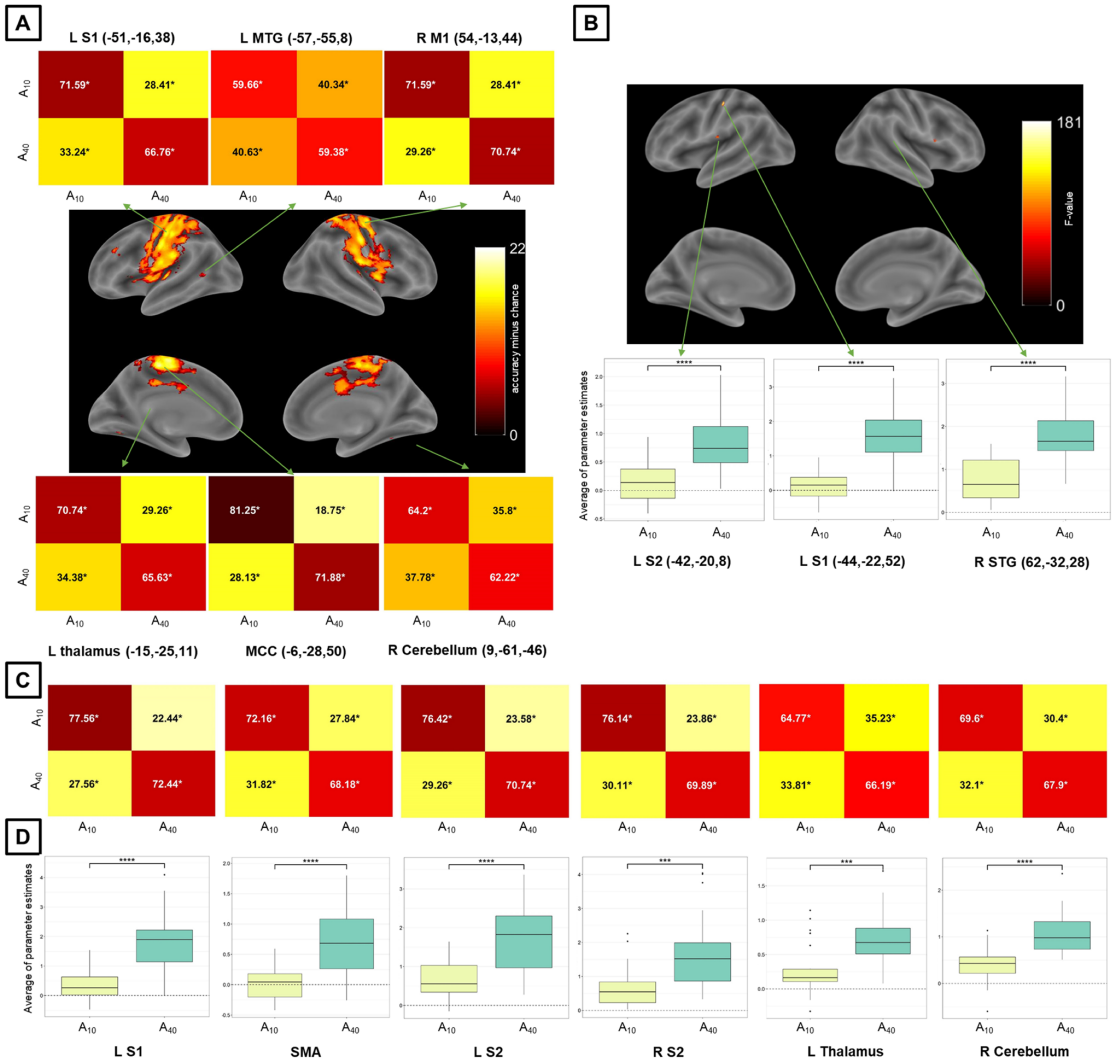
MVPA and univariate analysis results for the amplitude condition and Exp 2. (A) Whole-brain results for (A) MVPA (accuracy significantly above chance) and (B) univariate analysis (significant differential activation), both using a voxel-level inclusion threshold of p < 5 × 10^-5^, FWE (family-wise error)-corrected for multiple comparisons. ROI analysis results (C) for MVPA and (D) univariate analysis. Plots in (A) and (C) represent accuracies significantly different from chance. Plots in (B) and (D) represent significant main effects of amplitude in activation and post-hoc significant pairwise differences. Asterisks in MVPA results represent accuracies significantly different from chance (50%), corrected for multiple comparisons using the Bonferroni method and bootstrapping (10^5^ permutations) across subjects. Asterisks in univariate analysis results represent significant differences across activation estimates, corrected for multiple comparisons (Sidak method – ****p < 0.0001, ***p < 0.001). For MVPA confusion matrices, numbers in black (white) are higher (lower) than chance. S1/S2 = primary/secondary somatosensory cortex, MTG = middle temporal gyrus, M1 primary motor cortex, MCC = midcingulate cortex, STG = superior temporal gyrus, SMA = supplementary motor area, L/R = left/right.

For Exp 2 (which included two amplitude levels), whole-brain MVPA revealed a large brain network encoding amplitude levels of passive finger movement, which included the bilateral sensorimotor network (including S1 and S2, M1, and SMA) (Fig. 3A and Table 3). Whole-brain univariate analysis, for the same statistical threshold, showed differential activation across amplitude conditions in the contralateral S1 and S2, and the ipsilateral STG (Fig. 3B and Table 3). The activation estimates in these regions increased with amplitude. MVPA for Exp 2 showed high sensitivity, despite the strict statistical threshold compared to the analysis for amplitude in Exp 1. MVPA ROI analysis for Exp 2 further showed that all analyzed regions encode amplitude levels (Fig. 3C). For this MVPA ROI analysis in Exp 2, we also report accuracy confusion matrices for amplitude using a larger ROI (Supplementary Fig. S9A) and with scaling (Supplementary Fig. S11A), alongside the original values. Univariate ROI analysis also showed that all analyzed regions exhibited differential activation across amplitude levels (Fig. 3D). Stability was acceptable for amplitude in Exp 2 (Supplementary Fig. S1A, bottom row). Axial slices for whole-brain maps for amplitude in Exp 2 obtained with MVPA and univariate analysis are shown in Supplementary Figure S3.

**Table 3.** Whole-brain MVPA and univariate results for the amplitude condition and Exp 2.

			MNI coordinates		
Region label	Extent (voxels)	Statistics	x	y	z
*MVPA results*					
L MCC	6400	22.78	-6	-28	50
L postcentral gyrus (L S1)		19.33	-51	-16	38
R postcentral gyrus (R S1)		19.25	54	-13	44
L Thalamus	33	16.23	-15	-25	11
R Cerebellum (IX)	86	15.52	9	-61	-46
L middle temporal gyrus	20	13.81	-57	-55	8
R cerebellum (VI)	68	13.81	30	-52	-25
L IFG (p. Triangularis)	17	13.30	-39	29	29
L Fusiform Gyrus	11	12.57	-27	-64	-1
*Univariate analysis results*					
L insula lobe (L S2)	50	174.68	-42	-20	8
L postcentral gyrus (L S1)	25	181.72	-44	-22	52
R supramarginal gyrus (R SMG)	10	155.71	62	-32	28

Only clusters containing 10 or more voxels are reported. The statistics column corresponds to accuracy minus chance for MVPA results and to F-value to univariate analysis results. Brain regions were labeled through the Anatomy Toolbox and after visual inspection (between parentheses).

### 3.2 Velocity

#### 3.2.1 Experiment 1

For Exp 1 (which included three velocity levels), neither the whole-brain MVPA nor the whole-brain univariate analysis led to surviving clusters related to velocity of passive finger movement. Neither the MVPA nor the univariate ROI analysis showed significant results for velocity in Exp 1 (Supplementary Fig. S4). For the MVPA ROI analysis in Exp 1, we also report accuracy confusion matrices for velocity using a larger ROI (Supplementary Fig. S8B) and with scaling (Supplementary Fig. S10B), alongside the original values. There was no acceptable stability for velocity in Exp 1 (Supplementary Fig. S1B, top row).

#### 3.2.2 Experiment 2

**Fig. 4.**
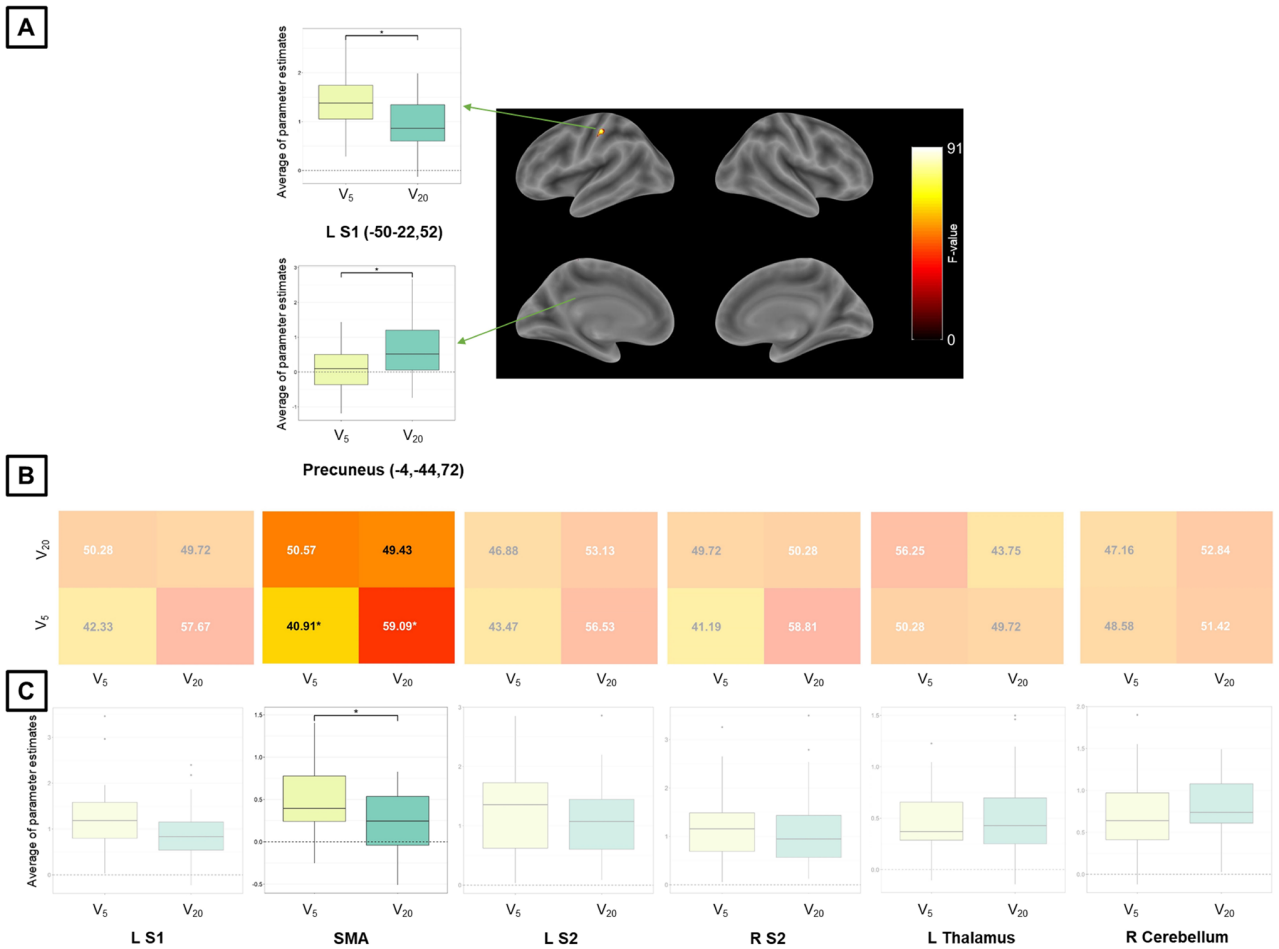
MVPA and univariate analysis results for the velocity condition and Exp 2. (A) Whole-brain results for univariate analysis (significant differential activation), using a voxel-level inclusion threshold of p < 0.05, FWE (family-wise error)-corrected for multiple comparisons. ROI analysis results (B) for MVPA and (C) univariate analysis. The non-transparent plot in (B) represents accuracies significantly different from chance. The non-transparent plot in (C) represents significant main effects of amplitude in activation and a post-hoc significant pairwise difference. The asterisks in the MVPA result represents accuracy significantly different from chance (50%), corrected for multiple comparisons using the Bonferroni method and bootstrapping (10^5^ permutations) across subjects. Asterisks in univariate analysis results represent significant differences, corrected for multiple comparisons (Sidak method – *p < 0.05). For MVPA confusion matrices, numbers in black (white) are higher (lower) than chance. S1/S2 = primary/secondary somatosensory cortex, SMA = supplementary motor area, L/R = left/right.

For Exp 2 (which included two velocity levels), whole-brain MVPA resulted in no surviving clusters for velocity of passive finger movement. Whole-brain univariate analysis showed, for the same statistical threshold, greater activity in the contralateral S1 for a lower compared to a higher velocity, as well as lower activity in the precuneus for a higher compared to a lower velocity (Fig. 4A and Table 4). MVPA and univariate ROI analysis for Exp 2 showed that the SMA was the only region, among the ones analyzed, to both encode (Fig. 4B) and exhibit differential activation (Fig. 4C) for velocity. For this MVPA ROI analysis in Exp 2, we also report accuracy confusion matrices for velocity using a larger ROI (Supplementary Fig. S9B) and with scaling (Supplementary Fig. S11B), alongside the original values. There was no acceptable stability for velocity in Exp 2 (Supplementary Fig. S1B, bottom row). Axial slices for whole-brain maps for velocity in Exp 2 obtained with univariate analysis are shown in Supplementary Figure S5.

**Table 4.** Whole-brain univariate results for the velocity condition and Exp 2.

			MNI coordinates		
Region label	Extent (voxels)	F-value	x	y	z
L postcentral gyrus (L S1)	79	91.03	-50	-22	52
L precuneus	24	64.64	-4	-44	72

Only clusters containing 10 or more voxels are reported. Brain regions were labeled through the Anatomy Toolbox and after visual inspection (between parentheses).

### 3.3 Direction

#### 3.3.1 Experiment 1

**Fig. 5.**
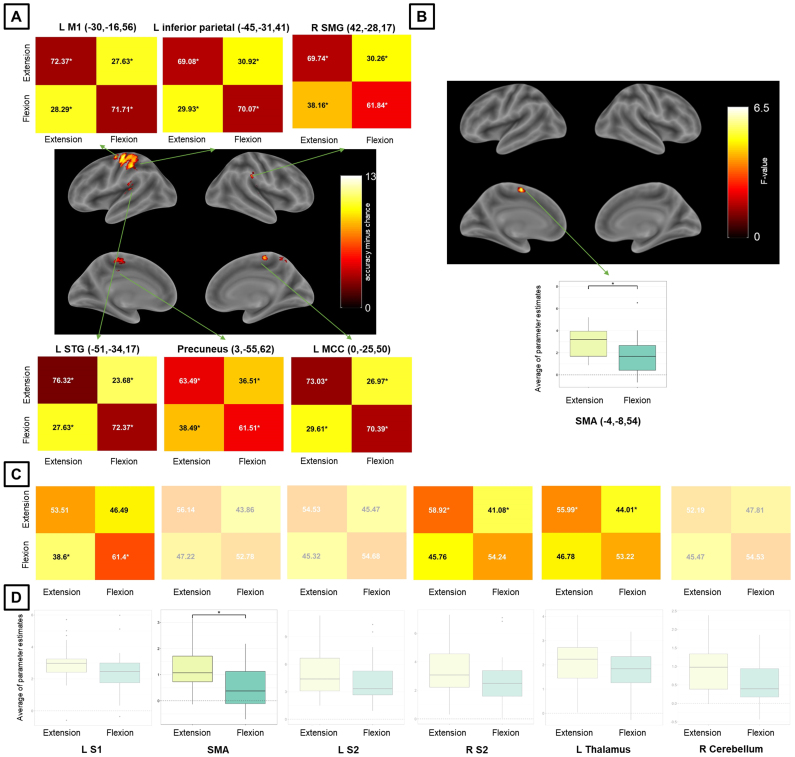
MVPA and univariate analysis results for the direction condition and Exp 1. Whole-brain results for (A) MVPA (accuracy significantly above chance) and (B) univariate analysis (significant differential activation), both using a voxel-level inclusion threshold of p < 0.05, FWE (family-wise error)-corrected for multiple comparisons. ROI analysis results (C) for MVPA and (D) univariate analysis. Non-transparent plots in (A) and (C) represent accuracies significantly different from chance. Non-transparent plots in (B) and (D) represent significant main effects of amplitude in activation and post-hoc significant pairwise differences. Asterisks in MVPA results represent accuracies significantly different from chance (50%), corrected for multiple comparisons using the Bonferroni method and bootstrapping (10^5^ permutations) across subjects. Asterisks in univariate analysis results represent significant differences across activation estimates and are corrected for multiple comparisons (Sidak method – *p < 0.05). For MVPA confusion matrices, numbers in black (white) are higher (lower) than chance. S1/S2 = primary/secondary somatosensory cortex, SMG = supramarginal gyrus, STG = superior temporal gyrus, M1 = primary motor cortex, MCC = midcingulate cortex, SMA = supplementary motor area, L/R = left/right.

For Exp 1, whole-brain MVPA revealed a brain network encoding direction levels of passive finger movement, which included contralateral S1, M1, and STG, ipsilateral SMG, and MCC and precuneus (Fig. 5A and Table 5). Whole-brain univariate analysis, for the same statistical threshold, showed higher activation for extension than for flexion in the SMA (Fig. 5B and Table 5). Whole-brain MVPA was more spatially sensitive than univariate analysis for direction of passive movement. MVPA ROI analysis for Exp 1 further showed that, in addition to contralateral S1, the ipsilateral S2 and thalamus also encode direction levels (Fig. 5C). For this MVPA ROI analysis in Exp 1, we also report accuracy confusion matrices for direction using a larger ROI (Supplementary Fig. S8C) and with scaling (Supplementary Fig. S10C), alongside the original values. Univariate ROI analysis showed the same result observed for whole-brain analysis (Fig. 5D). There was no acceptable stability for direction in Exp 1 (Supplementary Fig. S1C, top row). Axial slices for whole-brain maps for direction in Exp 1 obtained with MVPA and univariate analysis are shown in Supplementary Figure S6.

**Table 5.** Whole-brain MVPA and univariate results for the direction condition and Exp 1.

			MNI coordinates		
Region label	Extent (voxels)	Statistics	x	y	z
*MVPA results*					
L precentral gyrus (L M1)	581	13.50	-30	-16	56
L inferior parietal lobule		12.94	-45	-31	41
L MCC	99	9.44	0	-25	50
R precuneus	17	9.33	3	-55	62
Location not in atlas	12	8.54	-39	-40	26
L superior temporal gyrus (L STG)	58	8.25	-51	-34	17
R heschls gyrus (R STG)	14	8.17	42	-28	17
R supramarginal gyrus	12	7.77	57	-31	35
*Univariate analysis results*					
L SMA	133	6.54	-4	-8	54

Only clusters containing 10 or more voxels are reported. The statistics column corresponds to accuracy minus chance for MVPA results and to F-value to univariate analysis results. Brain regions were labeled through the Anatomy Toolbox and after visual inspection (between parentheses).

#### 3.3.2 Experiment 2

**Fig. 6.**
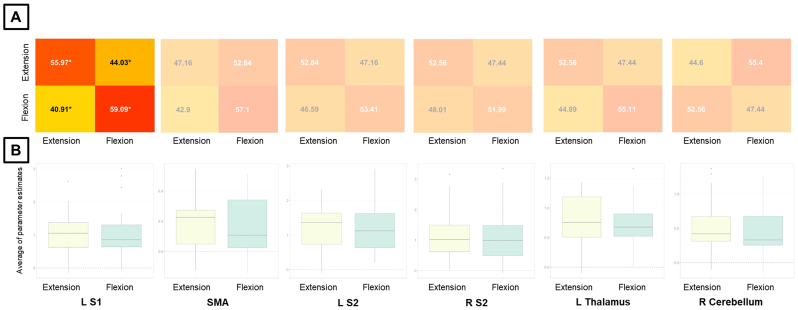
MVPA and univariate analysis results for the direction condition and Exp 2. ROI analysis results (A) for MVPA and (B) univariate analysis. The non-transparent plot in (A) represents accuracies significantly different from chance. Asterisks in MVPA results represent accuracies significantly different from chance (50%), corrected for multiple comparisons using the Bonferroni method and bootstrapping (10^5^ permutations) across subjects. For MVPA confusion matrices, numbers in black (white) are higher (lower) than chance. S1/S2 = primary/secondary somatosensory cortex, SMA = supplementary motor area, L/R = left/right.

For Exp 2, neither the whole-brain MVPA nor the whole-brain univariate analysis led to surviving clusters for the direction of passive finger movement. MVPA ROI analysis for Exp 2 showed that the contralateral S1 was the only region to encode direction levels (Fig. 6A). For this MVPA ROI analysis in Exp 2, we also report accuracy confusion matrices for direction using a larger ROI (Supplementary Fig. S9C) and with scaling (Supplementary Fig. S11C), alongside the original values. There were no significant results for univariate ROI analysis (Supplementary Fig. 6B) and no acceptable stability for direction in Exp 2 (Supplementary Fig. S1C, bottom row).

### 3.4 Comparison of significant voxel counts across conditions for MVPA and univariate analysis

In an exploratory analysis, we compared voxel counts within regions of interest that distinguished between levels of kinematic features across conditions, using both MVPA and univariate analyses (Table 6). For the MVPA, voxel counts were obtained from subject-specific whole-brain accuracy-minus-chance maps using a threshold of 10%. For the univariate analysis, voxel counts were derived from subject-specific maps from one-way ANOVAs across kinematic features levels. Overall, MVPA revealed significant differences across conditions in voxel counts within all examined regions, following a consistent pattern of Amplitude > Direction > Velocity (except in the thalamus, where voxel counts for Direction and Velocity did not significantly differ). In contrast, univariate analyses generally showed no differences in voxel counts across conditions, with the exception of the left postcentral gyrus, where the Amplitude condition yielded a significantly greater number of voxels compared to the Direction condition.

**Table 6.** Number of voxels within regions of interest that discriminated between levels of kinematic features across conditions, based on multivariate (MVPA) and univariate analyses.

Region	Mean ± standard deviation			Comparisons		
	Amplitude	Velocity	Direction	Amplitude–velocity	Amplitude–direction	Velocity–direction
MVPA						
Precentral L	613 ± 64	150 ± 15	298 ± 31	z = 9.53, p < 0.0001 *	z = 4.89, p < 0.0001 *	z = -4.64, p < 0.0001 *
Supplementary motor area L	328 ± 38	91 ± 11	151 ± 18	z = 7.78, p < 0.0001 *	z = 4.73, p < 0.0001 *	z = -3.05, p = 0.006 *
Postcentral L	841 ± 87	175 ± 18	400 ± 41	z = 10.73, p < 0.0001 *	z = 5.08, p < 0.0001 *	z = -5.66, p < 0.0001 *
Supramarginal L	237 ± 27	54 ± 6	109 ± 13	z = 9.02, p < 0.0001 *	z = 4.72, p < 0.0001 *	z = -4.31, p < 0.0001 *
Cerebellum R (lobule VI)	218 ± 27	54 ± 8	84 ± 10	z = 8.31, p < 0.0001 *	z = 5.66, p < 0.0001 *	z = -2.58, p = 0.026 *
Thalamus L (ventral posterolateral nucleus)	19 ± 4	4.7 ± 0.9	6 ± 1	z = 5.02, p < 0.0001 *	z = 4.14, p = 0.0001 *	z = -0.92, p = 0.6
Secondary somatosensory region L	57 ± 8	11 ± 2	26 ± 4	z = 8.38, p < 0.0001 *	z = 4.10, p = 0.0001 *	z = -4.30, p = 0.0001 *
Secondary somatosensory region R	50 ± 7	12 ± 2	22 ± 4	z = 6.79, p < 0.0001 *	z = 3.72, p = 0.0006 *	z = -2.90, p = 0.010 *
Univariate analysis						
Precentral L	410 ± 77	329 ± 76	180 ± 41	-	-	-
Supplementary motor area L	249 ± 68	204 ± 58	256 ± 58	-	-	-
Postcentral L	799 ± 170	427 ± 117	265 ± 65	z = 1.81, p = 0.17	z = 3.39, p = 0.0020 *	z = 1.30, p = 0.4
Supramarginal L	187 ± 44	99 ± 31	225 ± 63	-	-	-
Cerebellum R (lobule VI)	107 ± 20	73 ± 25	96 ± 24	-	-	-
Thalamus L (ventral posterolateral nucleus)	0	1.22	0	-	-	-
Secondary somatosensory region L	111 ± 25	79 ± 22	125 ± 32	-	-	-
Secondary somatosensory region R	98 ± 29	27 ± 10	85 ± 28	-	-	-

For the MVPA, voxel counts were derived from subject-specific whole-brain accuracy maps (classification accuracy above chance) using a threshold of 10%. For the univariate analysis, voxel counts were based on subject-specific maps from one-way ANOVAs across the levels of kinematic features.

Note: Means and standard deviations refer to the number of voxels within each region and condition across subjects. L/R = left/right. Asterisks indicate significant post-hoc pairwise comparisons. Hyphens denote where the zero-inflated generalized linear mixed model yielded non-significant results.

## 4 Discussion

We provide evidence for the brain regions that respond to different kinematic elements of passive finger movement using fMRI data from neurotypical subjects. In two experiments, we investigated how kinesthesia is encoded in the brain by using an MRI-compatible robot to provide subjects with systematic finger stimulation. Our analyses consisted of univariate and multivariate approaches, performed separately for each kinematic feature and using a model that accounts for the influence of stimulus duration. This model mitigates the false positive rates regardless of whether neural activity scales with stimulus duration. Previous research on muscle spindles and skin receptors in kinesthesia has suggested that proprioception can be dissociated into position, velocity, and direction senses (Proske & Gandevia, 2009). Therefore, such evidence has led to the hypothesis that kinematic features might also be spatially dissociated in the brain. We confirmed previous findings that a number of somatosensory and motor regions are related to the amplitude and direction of passive finger movement. In contrast, we observed fewer brain regions associated with the perception of velocity (Table 6).

### 4.1 Interpretation of univariate and multivariate results in fMRI

Univariate and multivariate approaches, which leverage the high spatial resolution of fMRI, can be utilized to investigate brain regions involved in psychological or physical conditions. (Although both approaches could be considered *multivariate* in principle, since univariate analysis involves estimating one weight per regressor and multivariate analysis one weight per voxel, we retain the standard nomenclature for consistency with the literature and clarity of comparison.) In univariate analysis, the BOLD time course in each voxel is fit to a predefined model for a condition of interest, and condition-related activity is inferred from differences in mean activation. Such differences suggest that neural responses at the voxel level are distinguishable, even after accounting for subject-level variability. However, this approach may be insensitive to neural processes that vary across neighboring voxels or when subject-level variability is high (Davis et al., 2014; Jimura & Poldrack, 2012). In contrast, multivariate approaches capture differences in the spatial configuration of activation across voxels, even when mean activation is equivalent across conditions, with these differences arising from heterogeneity in voxel responses. This approach employs a machine-learning algorithm to train a model that classifies patterns as corresponding to psychological or physical conditions. The model’s ability to successfully classify these conditions indicates that discriminative information about them is present in the specific brain region. MVPA is recognized for its greater sensitivity in mapping functional correlates of brain regions compared to univariate methods (Coutanche, 2013; Davis & Poldrack, 2013; Kriegeskorte et al., 2006; Norman et al., 2006). This is largely because MVPA can detect the distributed coding of information, whereas univariate analysis primarily captures single-voxel engagement in task conditions (Jimura & Poldrack, 2012).

It is therefore crucial to properly interpret the different outcomes obtained from univariate and multivariate approaches. A significant univariate result is typically taken to indicate higher activation in a brain region, reflecting greater processing or resource utilization (Coutanche, 2013). Conversely, MVPA may reveal reliable condition-related information embedded in distributed patterns of voxel activity that may not be captured by mean activation differences (Davis et al., 2014). Importantly, MVPA results should not be overinterpreted as evidence for neural representational complexity, i.e., that the pattern of activity necessarily encodes multiple distinct properties (Davis et al., 2014). For example, a region showing no univariate result but significant multivariate information suggests that activation variability due to a condition occurs in different directions across distributed voxels (Harrison & Tong, 2009). In practice, the neural interpretation of such a multivariate-only effect is that sub-populations of neurons encode conditions differently, although their averaged activity cancels out. In other words, condition-specific information is present in the region, but the activation direction (above or below baseline) is not consistent across participants. Conversely, the presence of univariate but not multivariate effects indicates robust mean-level activation differences across conditions, but with unreliable condition-related differences in distributed voxels patterns. The heightened sensitivity of MVPA is often attributed to its ability to detect heterogeneous spatial patterns that remain undetectable to univariate approaches (Coutanche, 2013; Davis et al., 2014).

Our univariate analysis revealed that certain regions showed unidirectional associations between activation and levels of passive movement features. In contrast, our MVPA identified specific regions where condition-related patterns of activation were detectable across voxels, even when mean activity did not differ across conditions. In our study, condition-related differences were tested with MVPA with leave-one-run-out cross-validation, which evaluates voxel patterns within individual subjects. Sets of voxels identified as carrying condition-specific patterns were then tested against the null hypothesis at the group level. Although MVPA is generally more sensitive than univariate analysis for detecting condition-related information, the resulting patterns can exhibit substantial between-subject variability. Indeed, our complementary leave-one-subject-out cross-validation analysis showed that the findings—except for the Amplitude condition in Exp 2—had limited generalization across subjects. The differences in sensitivity between these approaches may arise from several factors: (i) inference, as univariate analysis tests the reliability of effects, whereas MVPA evaluates the separability of patterns across conditions; (ii) feature selection, which can rely on univariate analysis to reduce noise before applying multivariate analysis; (iii) number of subjects, which generally improves power in univariate analyses but does not always increase classification accuracy in MVPA; and (iv) preprocessing, which enhances signal quality in univariate analysis but constrains model complexity in MVPA. To minimize methodological discrepancies, we applied identical preprocessing steps and GLM estimation to both analyses. Nevertheless, the inherent differences in the analytical frameworks make them not directly comparable. By presenting results from both approaches, we illustrate how univariate and multivariate analyses can yield distinct but complementary insights into the neural correlates of kinematic passive movement.

### 4.2 Amplitude of passive finger movement

We investigated brain regions associated with amplitude of passive finger movement. We confirmed previous findings of a network comprised of sensorimotor and subcortical regions associated with amplitude of passive finger movement and other body parts in humans (Boscolo Galazzo et al., 2014; Francis et al., 2009; Jaeger et al., 2014; Mima et al., 1999; Szameitat et al., 2012; Weiller et al., 1996). Overall, univariate analysis confirmed that activity in these regions increases with amplitude, concordant with studies on active movement (Shirinbayan et al., 2019) and afferent firing rates (Edin, 1992; Knibestöl, 1975). Compared to other kinematic features, we associated amplitude of passive movement with strong univariate differences and widespread discriminative information in multivariate analysis (Table 6). Limb position is first detected by muscle spindles (Goodwin et al., 1972) and skin receptors (Edin, 1992) and this information is then carried to the brain. Static discharges that proportionally change according to stretch of muscle spindles (Roll & Vedel, 1982). Stronger cortical responses to amplitude than velocity are congruent with the idea that sense of position relies heavily on spindle inputs. Although primary endings respond to both length and velocity changes, secondary endings are selectively sensitive to length (Matthews, 1974; Proske & Gandevia, 2009), which may underlie the greater univariate amplitude effects. As an alternative interpretation, during passive movement, the final position is unknown and the expectation of position is continuously updated by proprioception until the movement ends. Greater proprioceptive updating of positional expectation would, therefore, be required for estimating larger displacements during passive movement. Our univariate results suggest that activation in the somatosensory cortex scales with proprioceptive inputs from passive finger movement, while our MVPA results adds evidence of amplitude-related differences that can be found in distributed voxel activity in this region.

We observed contralateral S1 activity associated with amplitude of passive finger movement in Exps 1 and 2 (Figs. 2 and 3), which is consistent with previous studies on passive and active movement (Boscolo Galazzo et al., 2014; Guzzetta et al., 2007; Lolli et al., 2019, 2021; Shirinbayan et al., 2019; Weiller et al., 1996; Zhavoronkova et al., 2017). S1 activity was also observed in early studies of cortical recordings in nonhuman primates during passive finger movement (Gardner & Costanzo, 1981). In Exp 2, MVPA further revealed ipsilateral S1 discriminative information related to amplitude (Fig. 3A). The effects in S1 are consistent with its established role in receiving proprioceptive and somatosensory input that contributes to finger localization during passive movement (Mountcastle et al., 1992; Nurmi et al., 2018; Shirinbayan et al., 2019; Yousry et al., 1997). We also observed bilateral S2 involvement: for MVPA in Exp 1 (Fig. 2A, C) and for both MVPA and univariate analysis in Exp 2 (Fig. 3). S2 has been associated with passive finger movement (Dueñas et al., 2018; Mima et al., 1999), but less sensitive than S1 (Backes et al., 2000; Nelson et al., 2004; Nurmi et al., 2018). While S1 receives rather simple peripheral sensory information, S2 may be related to higher aspects of sensory processing and retention of relevant afferent aspects in working memory (Lemus et al., 2010). The presence of amplitude-related effects in S2 only for MVPA in Exp 1 (Fig. 2C) suggests that amplitude-related information in S2 is more spatially distributed than in S1.

We observed that the SMA, the contralateral SMG, the posterior part of the STG, and the contralateral M1 were also involved in the amplitude of passive finger movement (Figs. 2 and 3). The SMA, which contributes to movement preparation, planning, and control (Tanji, 1994), has been reported to be associated with passive movement (Alary et al., 1998; Carel et al., 2000; Guzzetta et al., 2007; Nurmi et al., 2018; Weiller et al., 1996). As reported in other studies on active and passive movement, we confirmed that SMA activation was more dominant in the contralateral hemisphere (Pool et al., 2013; Zhavoronkova et al., 2017). It has been suggested that the SMA receives information from the somatosensory system (Cadoret & Smith, 1997) and its activation (as well as the contralateral S1) is lower during passive compared to active movement (Lolli et al., 2021; Mima et al., 1999; Zhavoronkova et al., 2017). This pattern is consistent with the proposal that the SMA is more responsive to afferent input during active than passive movement and may contribute to differentiating intentional and involuntary movement (Mima et al., 1999). In a different vein, the SMG, located in the inferior parietal lobule and with strong connections with somatosensory regions, processes proprioceptive information (Ben-Shabat et al., 2015), and is involved in spatial perception (Bjoertomt et al., 2009). In addition, M1 is known to be primarily involved in motor execution of body parts. Consistent with our results, single-cell recordings from non-human primates (Schwartz, 1992), and estimation of BOLD activity (Shirinbayan et al., 2019) showed that M1 is associated with movement amplitude, but not velocity (Section 3.2). Interestingly, while M1 shows little response to tactile stimulation, it is active during both active and passive movement (Goldring & Ratcheson, 1972). Therefore, M1 is thought to be involved in proprioceptive feedback independent of skin stimulation (Jansma et al., 1998).

Besides the cortical regions, we also observed that the thalamus and the ipsilateral cerebellum were involved in the amplitude of passive finger movement (Fig. 2 and 3). Notably, in Exp 1, the thalamus and ipsilateral cerebellum, together with S1, were associated with amplitude only in the univariate analysis (Fig. 2D). This suggests that their contribution is reflected primarily in mean-level activation differences rather than in discriminative multivoxel patterns. The thalamus has been implicated in both active and passive movement, predominantly in the contralateral hemisphere (Boscolo Galazzo et al., 2014; Shirinbayan et al., 2019), and it sends direct projections to the somatosensory cortex (Viaene et al., 2011). More specifically, the thalamic ROI corresponds to the ventral posterolateral nucleus of the thalamus, a key relay area for the transmission of somatosensory information, such as touch and proprioception, from the spinothalamic tracts to S1 (Yoshita & Yamada, 2006). Similarly, ipsilateral cerebellar responses to passive movement have been documented (Boscolo Galazzo et al., 2014; Zhavoronkova et al., 2017). The cerebellar ROI corresponds to lobule VI of the cerebellum, which is associated with fine motor control and simple finger movement tasks (Manto et al., 2012). In the context of passive movement, processing of relevant features in the cerebellum is likely mediated by afferent input from brainstem pathways that project to both the cerebellum and the cortex (the latter via the thalamus) (Landgren & Silfvenius, 1971; Proske & Gandevia, 2009).

### 4.3 Velocity of passive finger movement

We also examined brain regions associated with the velocity of passive finger movement. We observed that sensorimotor network regions (contralateral S1 and SMA) showed a negative relationship between activation and velocity, whereas the precuneus showed a positive relationship with velocity. Compared to amplitude, fewer cortical brain regions were associated with velocity during passive movement (Fig. 4 and Table 6), which is consistent with previous evidence from spike train recordings in animals (Golub et al., 2014) and studies in humans using fMRI (Shirinbayan et al., 2019; Wenzel et al., 2014) during active movement. In addition, whole-brain univariate analysis yielded significant results in sensorimotor regions that, whereas whole-brain MVPA did not reveal discriminable condition-related patterns in these regions. This outcome aligns with the notion that univariate and multivariate analyses capture complementary aspects of neural activity.

Univariate activity in contralateral S1 and SMA was associated with velocity of passive movement, consistent with previous studies (Boscolo Galazzo et al., 2014; Dueñas et al., 2018; Mima et al., 1999; Szameitat et al., 2012). A study on active movement showed that the contralateral S1 is more specific for amplitude than for velocity, congruent with our hypothesis, whereas the SMA is equally involved in both amplitude and velocity (Shirinbayan et al., 2019). The peak coordinates for the main effect of velocity were more frontal than for amplitude. In contrast to S1, we observed no evidence for S2 involvement with velocity, similar to findings using nerve stimulation (Backes et al., 2000) or peripheral stimulation (Nelson et al., 2004). We found a negative relationship between velocity and activation in the contralateral S1 and SMA. This inverse relationship between activity and velocity, independent of stimulus duration, may be due to increased proprioceptive updating during slower trajectories of passive movement. Therefore, we suggest that proprioceptive afferent information during passive movement may depend on velocity, with slower trajectories carrying more proprioceptive information.

Some studies have reported the relationship between velocity of passive movement and brain activity based on oscillatory frequencies. Here, we argue that this movement pattern is fundamentally different from linear displacement and may not be directly comparable to our results. During passive linear displacement, the fingers start and end at different positions in space, and proprioception plays a more critical role in spatial localization than in the case of oscillatory stimulation. This experimental difference may be the reason why studies on passive and active movement reported that the sensorimotor cortex and cerebellum were strongly related to movement rate (Nurmi et al., 2018; Riecker et al., 2003; Taniwaki et al., 2003; Turner et al., 1998) while our results with linear velocity were less sensitive. Similar to our study, Shirinbayan et al. (2019) found less sensitive results for velocity of linear displacements during active movements compared to amplitude. It is also possible that, during rapid oscillatory movements, the neural mechanisms of proprioception for frequency and amplitude are blended. For instance, an early study showed that muscle vibration, initially perceived as movement during rapid oscillatory vibration, was perceived as a change in position at slower frequencies (McCloskey, 1973), with position and velocity dissociated in muscle spindles. In fact, it has been proposed that a moderate oscillation rate (and therefore dissociation of position and velocity) is optimal to generate the greatest activation in sensorimotor cortex (Bae et al., 2017). The univariate approach showed that the precuneus was positively related to the velocity of passive movement, consistent with previous findings implicating this region in passive movement (Jaeger et al., 2014; Sahyoun et al., 2004). The precuneus is part of a network that is activated by higher levels of internally-focused attention (Andrews-Hanna et al., 2014). Therefore, one possible interpretation is that faster passive movement induced heightened internally oriented attention and, consequently, elicited precuneus activity.

### 4.4 Direction of passive finger movement

Finally, we also investigated brain regions associated with the direction of passive finger movement, that is, extension or flexion. We found a set of regions in the somatosensory cortex associated with passive movement direction, roughly similar to the regions implicated in amplitude. Specifically, we found that contralateral S1 and M1, contrateral STG, ipsilateral S2, SMA, and thalamus were associated with passive movement direction (Figs. 5 and 6). In univariate analysis, SMA activity was higher during extension than flexion. Compared to other kinematic features, results regarding movement direction are less frequently reported, with some studies finding no effects (Dueñas et al., 2018; Shirinbayan et al., 2019).

The contralateral S1 related to the direction of passive movement was identified only by MVPA and not by the univariate approach. This suggests that direction-related information is carried in distributed voxel patterns rather than reflected in mean-level activation differences. Consistent with this interpretation, Hammer et al. (2016) suggested that directional tuning is best represented by large populations of neurons and Eisenberg et al. (2010) showed that directional tuning follows a complex spatial organization that may be lost in group univariate analyses. Previous evidence suggests that directional afferents are primarily based on skin receptors (Proske & Gandevia, 2009). We, therefore, speculate that afferent information about direction from skin receptors may have a more distributed cortical representation than amplitude, but further studies are needed to confirm this hypothesis. Clinically, when the proprioception is assessed, patients are typically asked to indicate the direction of a passive movement (usually, up or down) (Lincoln et al., 1998). Our results indicate that the amplitude of passive finger movement is more broadly represented in the brain, suggesting that this kinematic feature could be advantageously used to design more sensitive clinical assessments (Zbytniewska et al., 2021).

### 4.5 Methodological considerations

Here we would like to address some points related to the modeling strategy used in this study. Of all the combinations of experiments and kinematic features, only the data from Exp 2 for amplitude led to acceptable cross-subject stability (Supplementary Fig. S1A, bottom row), that is, the model trained with these data was reproducible across subjects. We argue that the increased sensitivity combined with the reduced number of levels may have been important for achieving acceptable stability. Furthermore, Exp 2 yielded results with higher sensitivity to velocity than Exp 1 (Supplementary Figs. S4 and 4), thus fewer levels may have contributed to improved sensitivity for studying the brain correlates of velocity. However, in contrast to amplitude and velocity, data from Exp 1 yielded more sensitive results for direction than Exp 2. This discrepancy may be because Exp 2 had fewer trials available to estimate run-specific betas than Exp 1.

Amplitude, velocity, and stimulus duration are inherently related, and varying one feature inevitably implies varying another. In block designs, varying velocity, for example, implies that data will be confounded with frequency or number of repetitions (Shirinbayan et al., 2019). In an event-related design, varying, for example, amplitude implies that velocity or stimulus duration will also vary (Turner et al., 2003). Therefore, it is desirable to control for stimulus duration when studying brain activation related to physical movement. Time-on-task is positively correlated with BOLD activation (Taylor et al., 2014; Yarkoni et al., 2009) and can confound the results (Mumford et al., 2015). While reaction time in cognitive neuroscience may carry relevant information (Pamplona et al., 2022; Woolgar et al., 2014), stimulus duration in physical stimulation may be of less physiological interest. Stimulus duration can also act as a confound in MVPA, that is, the classifier may detect brain regions that are associated not only with the condition of interest, but also with duration differences (Todd et al., 2013). In fact, MVPA is highly sensitive to any systematic differences across conditions (Woolgar et al., 2014), and thus addressing confounders is important to ensure reliable results and valid interpretations.

The first study to analyze the present dataset (Dueñas et al., 2018) estimated activation using variable epochs (Grinband et al., 2008) to account for stimulus duration in the model. This approach appropriately scales linearly the estimate when neural activation scales with stimulus duration, but leads to inflated error rates when activation does not scale with stimulus duration (Mumford et al., 2024), which may be fundamentally the case when characterizing brain regions that are intrinsically related to sensorimotor components of passive movement rather than simply stimulus duration. In the present study, we constructed the regressors with constant-duration boxcar functions modulated by stimulus duration, which accounts for stimulus duration and mitigates false positives regardless of whether neural activation scales with stimulus duration (Mumford et al., 2024). This methodological difference may explain the discrepancies in univariate results between the seminal study and ours. For instance, our study found a positive relationship between contralateral S1 activation and amplitude, whereas the previous study found a negative relationship. In the current study, the model accounted for stimulus duration and controlled for false positives whether or not activation scaled with duration. Our estimate is also unbiased, but exhibits high variance due to the degree of collinearity between stimulus duration and amplitude/velocity (Mumford et al., 2015). This reduces statistical power in univariate analysis, although the higher sensitivity of MVPA can still identify relevant condition-related information. In addition, our model assumed that neural activations in our study could potentially be related to stimulus duration at different levels for each condition, representing a full interaction model (Mumford et al., 2024). In a complementary analysis, we assessed whether assuming that neural activations relate to stimulus duration independent of condition would alter the trend of our results (Supplementary Fig. S7). In this alternative model, increased variance prevented significant inferences, though activation remained positively associated with amplitude and negatively associated with velocity (Supplementary Fig. S7A-D). A trend for higher activation during extension compared to flexion was also reproduced (Supplementary Fig. S7E). Therefore, our full-interaction model effectively accounts for stimulus duration regardless of whether brain activation scales with it, while retaining power to infer significant differences across condition levels in univariate analysis.

Exps 1 and 2 used slow and rapid event-related designs, respectively. Rapid (compared to slow) event-related designs reduce the efficiency of hemodynamic response function (HRF) estimation due to greater overlap between adjacent responses, which can increase the variance of the parameter estimates and consequently decrease sensitivity. On the other hand, rapid event-related designs are often preferred experimentally and analytically because they allow the inclusion of more trials and consequently more condition levels within a run. This likely explains the observed idiosyncrasies between univariate and multivariate approaches for amplitude in Exp 1. For instance, amplitude was associated with the S2 response in the MVPA but not in the univariate analysis, and with the thalamus and cerebellum in the univariate but not in the MVPA. In contrast, the more sensitive results for amplitude and velocity in Exp 2 were likely due to its improved estimation of the hemodynamic response compared to Exp 1.

We would like to note that the adopted steps in the classification approach were not chosen with the primary goal of accuracy optimization. One key factor is the ROI size, applied both in the ROI analysis and the post-hoc analysis of accuracy peaks in the whole-brain results. In the case of MVPA, the relatively small number of voxels (19 voxels) may have resulted in increased variance in accuracy estimates and reduced classifier stability. To provide a comprehensive view, we presented results for two ROI sizes: 5-mm and 8-mm radius (Supplementary Figs. S8 and S9). Generally, accuracy tended to be higher for a larger ROI size due to the greater number of voxels contributing to classification. However, ROI size was selected to prioritize the location specificity of functionally relevant regions, ensure consistency between univariate and multivariate analyses (by using the same ROI size), and standardize post-hoc analysis of whole-brain peaks, regardless of cluster size. By reporting results for different ROI sizes, we aim to facilitate comparisons with univariate analyses and demonstrate that higher accuracy can be achieved with larger ROIs. Another methodological factor, feature scaling, was also evaluated, and the results are presented alongside the original ones (Supplementary Figs. S10 and S11). Only minor differences in accuracy were observed between classifications performed with and without feature scaling. This is consistent with a previous report suggesting that scaling has little influence on classification when beta image maps are used as predictors, since these data are already somewhat scaled by the general linear model relative to each run (Hebart et al., 2015).

Finally, an alternative explanation for the negative association between activation and passive movement velocity may be due to the estimation procedure. Nurmi et al. (2018) reported that, in a block design, S1 activity gradually increased with movement rate, but plateaued or even decreased at higher rates. The authors observed that relatively lower rates better tracked the convolved HRF. Since both the univariate analysis and the MVPA in our study relied on the GLM, the results depend on how well the BOLD signal captures the assumed HRF. However, we argue that such a degradation of estimation may be less likely in an event-related design. Further studies may help clarify this point.

### 4.6 Limitations

The present study has several limitations. First, moving the index finger in opposition to the thumb is only one of a multitude of possible finger movements. Future MRI-compatible robotic solutions could incorporate more degrees of freedom to explore and characterize the kinaesthetics of passive movement for other hand movements. Second, on a methodological level, only four runs were available for the cross-validation procedure used to determine the performance of the predictive model, and a larger number of runs could have led to more robust results. Third, for training the model, we used a linear support vector machine (SVM) to separate classes. While non-linear models may sometimes capture more complex decision boundaries, linear SVMs are widely preferred in neuroimaging for their interpretability and reduced risk of overfitting. Fourth, we used ROIs based on coordinates previously reported in related literature (Boscolo Galazzo et al., 2014). Individualizing the definition of the ROIs, using either functional or anatomical strategies, would presumably lead to more sensitive ROI analyses (Pamplona et al., 2020). Fifth, a previous study showed that MVPA classification performance is improved for rapid event-related designs through running separate GLMs for each trial and combining all the other trials into nuisance regressors (Mumford et al., 2012). The GLM approach we used, which consisted of estimating all the regressors of interest simultaneously, is a common procedure, but leads to higher parameter estimation variability compared to separate GLMs. However, the same study concluded that both approaches produce similar classification performance when the design contains inter-stimulus intervals of at least 4 s on average, which was the case for Exp 1. Sixth, our methodology cannot rule out the possibility that participants moved their fingers in response to the robotic displacements, despite being instructed not to do so. While electromyography (EMG) could have quantified active movement, acquiring reliable EMG data within the MRI scanner environment is challenging due to compatibility constraints and susceptibility to electromagnetic interference (Hétu et al., 2013). The randomized combinations of amplitude and velocity likely reduced the risk of condition-specific confounds related to active movement. Nevertheless, future studies could benefit from incorporating MRI-compatible EMG to control for this possibility.

### 4.7 Conclusions

Here, we studied the association between brain regions and kinematic features of passive index finger movement, independently of each other. This was achieved through univariate and multivariate analyses, systematic robotically driven passive movement stimulation, and explicit control for stimulus duration, regardless of whether brain activation scaled with it. We found that a large network of sensorimotor and motor regions carried information about the amplitude of passive finger movements, whereas condition-related information for velocity and direction was less robust. The predominance of amplitude over other kinematic features suggests that brain activity related to passive movement may primarily reflect the measurement of finger position through muscle spindles and the continuous updating of expected position. Understanding how passive movement is translated into neural responses is essential for designing neurally guided passive movement therapy after brain injury. The findings presented here may help track neural reorganization and inform how neurorehabilitation can leverage neural representations of kinematic features to promote brain recovery.

## Supplementary Material

Supplementary Material

## Data Availability

All obtained results and scripts used for the data analysis are available on the public GitHub repository: https://github.com/gustavopamplona/MVPA_passive_movement.
